# Interventions Based on Biofeedback Systems to Improve Workers’ Psychological Well-Being, Mental Health, and Safety: Systematic Literature Review

**DOI:** 10.2196/70134

**Published:** 2025-09-12

**Authors:** Simão Ferreira, Matilde A Rodrigues, Catarina Mateus, Pedro Pereira Rodrigues, Nuno Barbosa Rocha

**Affiliations:** 1 RISE-Health Center for Translational Health and Medical Biotechnology Research Polytechnic University of Porto Porto Portugal; 2 Health Data Science Faculty of Medicine University of Porto Porto Portugal; 3 CINTESIS@RISE—Centre for Health Technologies and Services Research Faculty of Medicine University of Porto Porto Portugal; 4 MEDCIDS—Department of Community Medicine, Information and Decision Sciences Faculty of Medicine University of Porto Porto Portugal

**Keywords:** biofeedback, well-being, mental health, breathing techniques, occupational safety, occupational health

## Abstract

**Background:**

In modern, high-speed work settings, the significance of mental health disorders is increasingly acknowledged as a pressing health issue, with potential adverse consequences for organizations, including reduced productivity and increased absenteeism. Over the past few years, various mental health management solutions, such as biofeedback applications, have surfaced as promising avenues to improve employees’ mental well-being. However, most studies on these interventions have been conducted in controlled laboratory settings.

**Objective:**

This review aimed to systematically identify and analyze studies that implemented biofeedback-based interventions in real-world occupational settings, focusing on their effectiveness in improving psychological well-being and mental health.

**Methods:**

A systematic review was conducted following the PRISMA (Preferred Reporting Items for Systematic Reviews and Meta-Analyses) guidelines. We searched PubMed and EBSCO databases for studies published between 2012 and 2024. Inclusion criteria were original peer-reviewed studies that focused on employees and used biofeedback interventions to improve mental health or prevent mental illness. Exclusion criteria included nonemployee samples, lack of a description of the intervention, and low methodological quality (assessed using the Physiotherapy Evidence Database [PEDro] checklist). Data were extracted on study characteristics, intervention type, physiological and self-reported outcomes, and follow-up measures. Risk of bias was assessed, and VOSviewer was used to visualize the distribution of research topics.

**Results:**

A total of 9 studies met the inclusion criteria. The interventions used a range of delivery methods, including traditional biofeedback, mobile apps, mindfulness techniques, virtual reality, and cerebral blood flow monitoring. Most studies focused on breathing techniques to regulate physiological responses (eg, heart rate variability and respiratory sinus arrhythmia) and showed reductions in stress, anxiety, and depressive symptoms. Mobile and app-directed interventions appeared particularly promising for improving resilience and facilitating recovery after stress. Of the 9 studies, 8 (89%) reported positive outcomes, with 1 (11%) study showing initial increases in stress due to logistical limitations in biofeedback access. Sample sizes were generally small, and long-term follow-up data were limited.

**Conclusions:**

Biofeedback interventions in workplace settings show promising short-term results in reducing stress and improving mental health, particularly when incorporating breathing techniques and user-friendly delivery methods such as mobile apps. However, the field remains underexplored in occupational contexts. Future research should address adherence challenges, scalability, cost-effectiveness, and long-term outcomes to support broader implementation of biofeedback as a sustainable workplace mental health strategy.

## Introduction

### Background

In today’s fast-paced and demanding work environments, mental health disorders are increasingly recognized as a critical health concern, with potentially harmful effects on organizations [1] that are far-reaching and extend beyond individual well-being, decreasing productivity and heightening absenteeism [2,3]. Extensive literature supports the link between occupational stress, psychosocial factors, and an increased risk of depression, as well as alcohol and drug consumption. Work-related stress can trigger a domino effect, leading to a chain of negative consequences. Heavy workloads pile up, leaving employees drowning in a sea of tasks. Job satisfaction plummets, contributing to lackluster performance and a feeling of being physically present but mentally absent, also known as “presenteeism.” With stress lurking in the background, accidents and injuries become more frequent, turning the workplace into a potential danger zone [4-7]. According to the Organisation for Economic Co-operation and Development and European Union (EU) [8], mental health problems, such as stress, anxiety, and depression, affect approximately 84 million people in the EU, with 1 in 4 workers reporting exposure to risk factors that can negatively affect mental well-being. In 2022, the same report portrays a scenario where 1 in 2 young Europeans reported unmet needs for mental health care in spring 2022 [9]. In view of this, the EU Strategic Framework on Health and Safety at Work 2021-2027 adopted by the European Commission reinforces the fight against psychosocial risks [10]. It is of paramount importance to face these challenges by designing and implementing interventions that target the promotion of good mental practices and preventing illness [10].

In recent decades, various solutions for managing mental health in occupational settings have emerged, including cognitive behavioral therapy [11,12]; stress, coping, or mindfulness training [13-15]; problem-solving [16,17]; and acceptance and commitment therapy training [18,19], among others. In the same pace, there has been a surge in the introduction of new solutions designed to monitor and improve employee’s mental health [20,21], such as biofeedback applications [22].

Biofeedback emerges as a therapeutic technique aimed at facilitating the acquisition of skills in controlling a diverse range of physiological processes, encompassing muscle tension, heart rate (HR), stress levels, and brain wave activity, thereby fostering enhancements in mental health and overall well-being [23]. This methodology uses electronic apparatuses to gauge and present physiological responses, including muscle activity, skin temperature, HR, and brain wave patterns, which serve as feedback to guide individuals toward deliberate and purposeful alterations in their bodily functions [24]. Within a conventional biofeedback session, an individual is connected to sensors that monitor specific physiological responses, and the ensuing data are instantaneously visualized on a computer screen, granting valuable insights into their bodily reactions while enabling conscious adaptations to ameliorate their well-being [25]. For instance, if an individual encounters heightened stress levels, the biofeedback session could potentially expose an augmented HR and muscle tension, thus furnishing the individual with actionable information to consciously induce muscle relaxation and regulate their breath, ultimately reducing stress levels [26,27].

Despite the increasing popularity of physiological monitoring technologies such as electroencephalography, eye-tracking, and biosensing for fatigue [28] and stress management in occupational settings, biofeedback offers a distinct approach [29-31]. While physiological monitoring technologies passively measure and track bodily signals, biofeedback actively engages the individual using real-time feedback to enable self-regulation of physiological responses [27,32,33]. This unique characteristic of biofeedback—facilitating the conscious control of bodily processes—differentiates it from passive monitoring techniques.

In this context, biofeedback serves not only as a tool for measurement but also as an intervention aimed at improving mental health and well-being through active participation. This process is particularly valuable in occupational settings, where employees can be empowered to manage stress, anxiety, and other mental health challenges through real-time adjustments to physiological responses, such as HR, muscle tension, and respiration [34].

Wearable devices are used in several biofeedback scenarios such as rehabilitation for providing biofeedback on biomechanical or physiological body parameters, holding promise in enhancing outcomes for individuals [35]. However, there remains a need to establish the prevalent sensor configurations commonly used and to determine the specific biofeedback components used for various pathologies [36,37]. In areas such as stress and sleep monitoring, companies in the health and performance technology sector must engage with consumers and identify real-world needs to gain a competitive advantage. In addition, investing in rigorous research to substantiate the effectiveness of their products is paramount. In contrast, consumers seeking optimal value should exercise care when selecting such products, considering both their personal requirements and the strength of supporting evidence regarding product effectiveness. By aligning these considerations, both companies and consumers can contribute to the advancement and adoption of impactful health and performance technologies [38].

While there is some evidence about the effectiveness of biofeedback-based interventions with different populations, we must carefully address the traditional laboratory application of biofeedback techniques in real-world applications. Argent et al [39] published a use case about the relevance of real-world validation of systems using wearable exercise biofeedback platforms, emphasizing that differences between laboratorial environment and real-world settings should be carefully addressed. While this case study is specifically for machine learning, the authors emphasized the need to map and understand results of biofeedback systems in previous studies developed in real-world environment and occupational settings, as well as the methodology used. However, as far as we know, there is no systematic literature review of the feasibility and effectivity of biofeedback-based mental health management tools and interventions involving workers of different occupational settings. Ter Harmsel et al [40] analyzed the feasibility of bio cueing and ambulatory biofeedback in the treatment of emotion regulation difficulties in psychiatric and nonpsychiatric populations; however, their study did not specifically focus on occupational settings. Yu et al [27] developed a systematic literature review on biofeedback applications for nonmedical stress management, but without a specific focus on occupational settings. Kennedy-Metz and Parker [41] conducted a systematic review of biofeedback on real-time stress management intervention, limiting it to research that included biofeedback administered during the task; however, studies included were not limited to employees. None of these previous reviews have delved deeply into the analysis of the content and quality of the interventions.

### Objectives

This systematic review aimed to identify and analyze interventions based on biofeedback that were specifically designed to improve employees’ psychological well-being and mental health and that were tested in working settings. Our review focuses specifically on biofeedback as a self-regulation tool, distinct from other technologies, and its application within occupational safety and mental health management.

As biofeedback interventions are increasingly explored in other fields, we aim to fill the gap in the literature by systematically reviewing biofeedback-based interventions specifically designed for occupational settings for the purpose of improving employee mental health and psychological well-being, with the goal of understanding their effectiveness and potential to enhance workplace safety. The results will also be discussed in terms of potential features related to higher engagement, better adherence, and improved workspace safety.

## Methods

### Eligibility Criteria

This systematic review was focused on studies in which biofeedback interventions targeted employees and workers as the primary population. Both experimental and pilot studies involving a range of mental health disorders that could be addressed using biofeedback were considered. In addition, studies included a description of interventions aimed at preventing mental health disorders and improving well-being. Given the novelty of the research area, this review aimed to be as inclusive as possible, including experimental and observational studies. However, these studies had to be original and published in scientific peer-reviewed journals.

Studies were excluded based on the following criteria: (1) the sample did not consist of employees; (2) lack of a clear description of the intervention; (3) publication type was a literature review, commentary, or editorial; and (4) poor methodological quality, as assessed using the Physiotherapy Evidence Database (PEDro) checklist (Table S1 in Multimedia Appendix 1).

### Information Sources and Search Strategy

This systematic review was conducted in accordance with the PRISMA (Preferred Reporting Items for Systematic Reviews and Meta-Analyses) guidelines [42]. On March 11, 2024, the search for relevant articles published in the last 13 years (2012-2024) was conducted through independent searches in PubMed and on EBSCO databases.

The search focused on identifying articles that included keywords under the following general categories: mental health, biofeedback, and workplace. The keywords used for the search are described in Textbox 1 and Multimedia Appendix 1. The search combined the different keywords and Boolean terms and was limited to papers published in English in the last 13 years.

**Keywords used for the search.**

**Keyword family and specific search terms**

- Mental health and job performance AND: “fatigue” OR “lassitude” OR “mental health” OR “mental disorders” OR “stress” OR “mood disorders” OR “behavioral symptoms” OR “anxiety” OR “burnout” OR “absenteeism” OR “Job performance” OR “Performance at Work” OR “productivity” OR “efficiency” OR “Occupational Stress” OR “Job satisfaction” OR “Quality of working life”
- Biofeedback AND: “biofeedback” OR “bio-feedback” OR “feedback” OR “wearable” OR “wearable electronic devices” OR “monitoring, physiological” OR “clinical alarms” OR “outcome measures” OR “real time” OR “self-monitoring”
- Workplace: “job” OR “job site” OR “workplace” OR “work place” OR “worker” OR “employee” OR “occupation” OR “operators” or “Occupational”

### Data Collection Process

Search results were exported to EndNote (version 20; Clarivate) software for screening. After removing duplicates, 2 authors independently reviewed the titles and abstracts to identify eligible studies based on the specified inclusion and exclusion criteria (Textbox 2). Full texts of articles were then analyzed, and conflicts were resolved through consensus. Some disagreements between reviewers at each stage of the selection process were solved through discussion and brief meetings. Finally, the search results and study selection process are fully detailed in the PRISMA flow diagram including the reasons for study exclusion. In addition, as biofeedback interventions increasingly involve web-based and mobile health applications, we integrated elements from the CONSORT-EHEALTH (Consolidated Standards of Reporting Trials of Electronic and Mobile Health Applications and Online Telehealth) checklist to enhance reporting rigor and standardization [43].

Key data were independently charted by 2 reviewers using a structured extraction form. Extracted variables included citation details, country, intervention groups, participant population, type and description of the biofeedback intervention, outcome measures (including both questionnaires and physiological data), main results, and follow-up information. Any discrepancies during the charting process were resolved through discussion. We used a narrative synthesis approach to analyze and interpret the findings from the included studies. The extracted data were systematically organized into categories based on intervention types, outcome measures, and study populations. We then analyzed the content to identify common patterns, emerging trends, and knowledge gaps across the studies. This synthesis allowed us to highlight the effectiveness of different biofeedback approaches, variations in delivery methods, and the specific psychological and physiological outcomes reported.

**Inclusion and exclusion criteria.**

**Inclusion criteria**

- Population: employees or workers in any occupational setting (eg, corporate, health care, and industrial)
- Article type: original peer-reviewed experimental studies, quasi-experimental studies, observational studies, or pilot trials
- Intervention description: studies implementing biofeedback-based interventions targeting mental well-being, stress, or safety
- Setting: real-world workplace or occupational settings
- Outcomes: psychological well-being, stress reduction, anxiety management, and physiological markers (eg, heart rate variability and cortisol)
- Language: articles published in English
- Publication date: studies published between 2012 and 2024
- Methodological quality: assessed with acceptable scores on the Physiotherapy Evidence Database (PEDro) checklist

**Exclusion criteria**

- Population type: studies focusing on nonworking populations (eg, students, retirees, and unemployed individuals)
- Article type: reviews, editorials, commentaries, conference abstracts, or other nonoriginal studies
- Intervention description: biofeedback used exclusively for clinical or rehabilitation purposes unrelated to workplace mental health
- Setting: studies conducted exclusively in laboratory environments
- Outcomes: no relevant mental health or physiological outcomes reported
- Language: articles published in languages other than English
- Publication date: studies published outside this range (2012 to 2024)
- Methodological quality: studies with poor methodological quality based on the PEDro checklist

### Assessment of Methodological Quality

The quality and risk of bias of all identified studies were assessed. To this end, a list of assessment criteria was constructed using the PEDro checklist [44]. Two authors independently assessed and rated each study in relation to methodological rigor, selection, and reporting bias. Eligible studies were critically appraised by 2 independent reviewers.

### Visualization of Biofeedback Publications

VOSviewer [45] was used for graphical analysis and visualization of the dataset. Keywords were used to extract relevant topics from the dataset. VOSviewer’s mapping and clustering methods facilitated the creation of a 2D map, delineating the topics in terms of frequency, relatedness, and clustering. The representation of each topic was determined by its frequency of occurrence, visualized by the size of nodes and labels. Distinct clusters were formed and differentiated by color for clear visualization [46].

## Results

### Search Results

**Figure 1:**
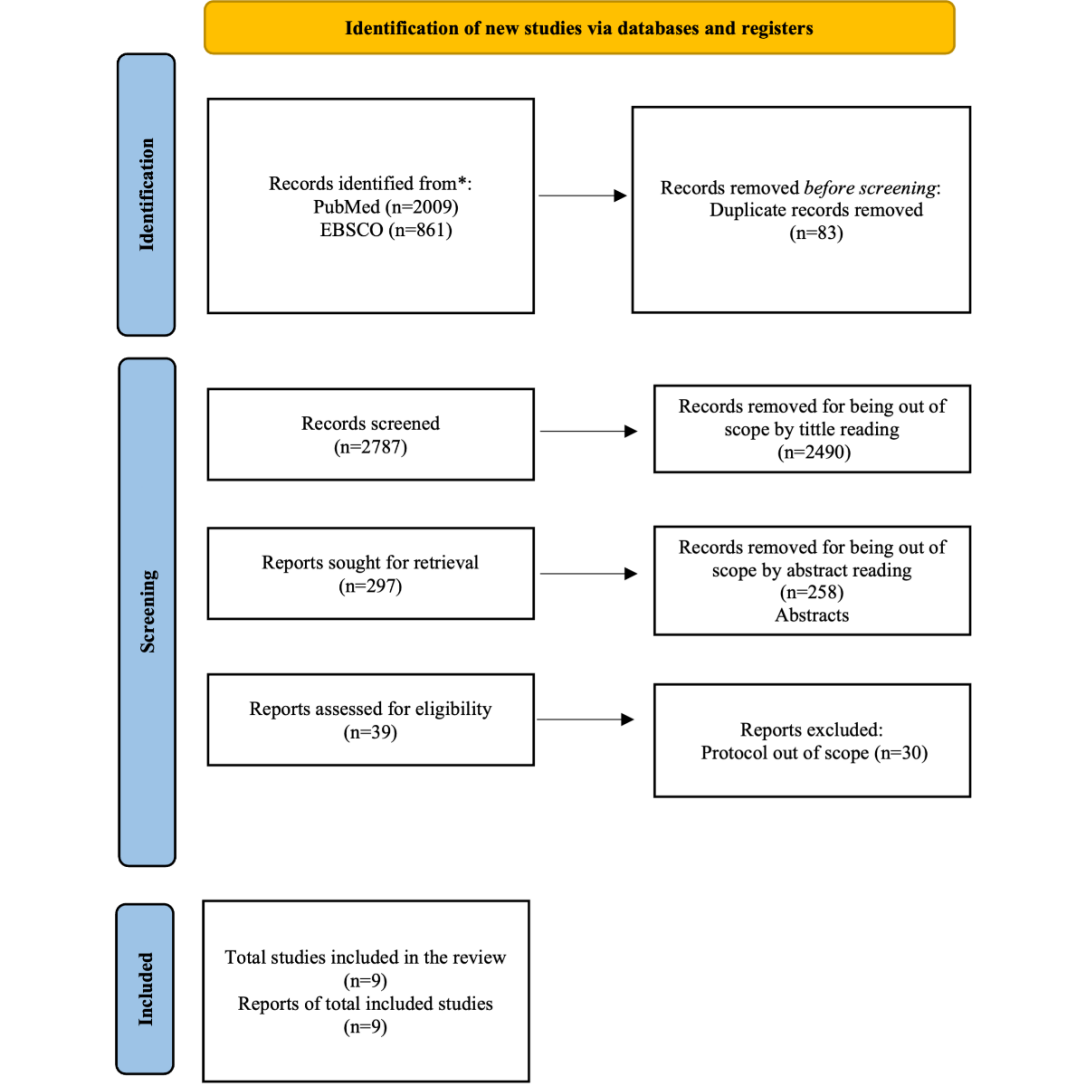
Flowchart of the systematic review following the PRISMA guidelines.

A total of 2787 articles were screened regarding the title only, resulting in 297 potential candidate publications. After reviewing the abstracts, 39 studies initially seemed relevant for the final content examination. However, after full-text retrieval and data extraction based on the relevance criteria outlined in the PRISMA flowchart, the number of studies pertinent for analysis was narrowed down to 9, as the remaining studies were deemed out of scope (Figure 1).

**Figure 2:**
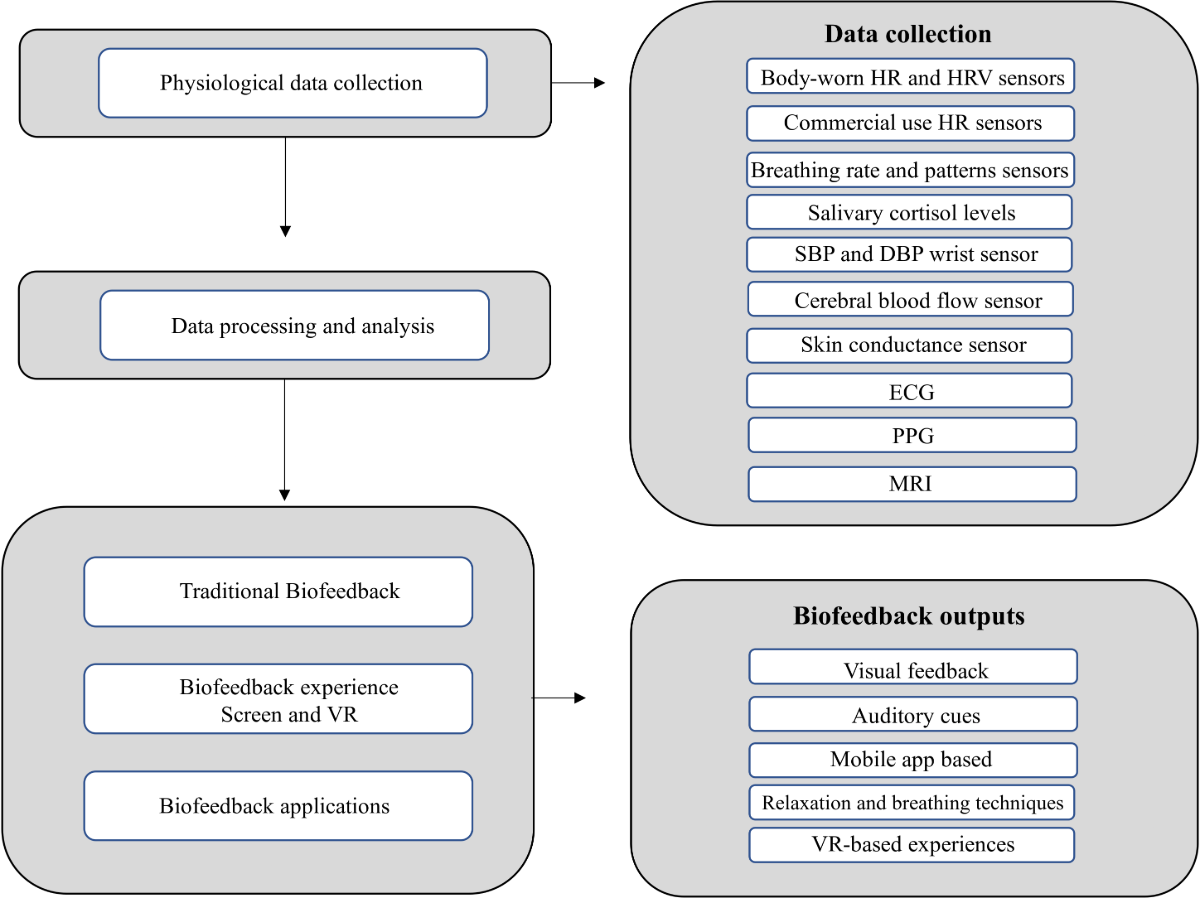
Process across all the selected studies from data collection to intervention models. ECG: electrocardiogram; HR: heart rate; HRV: heart rate variability; MRI: magnetic resonance imaging; PPG: photoplethysmography.

The obtained studies, published between 2012 and 2021, focused on interventions based on biofeedback or a combination of biofeedback with other interventions, specifically targeting workers or employees. Table 1 provides an overview of the studies, including the year of publication, sample size, intervention type, and results. Results denote that despite the various techniques, sensors, and approaches to physiological data treatment, most studies followed a similar path and series of events. Each study collected physiological data, processed and analyzed it, and provided feedback through various means, such as physical screens, applications, experiences, or stimuli, to help participants engage with the intended intervention (Figure 2).

**Figure 3:**
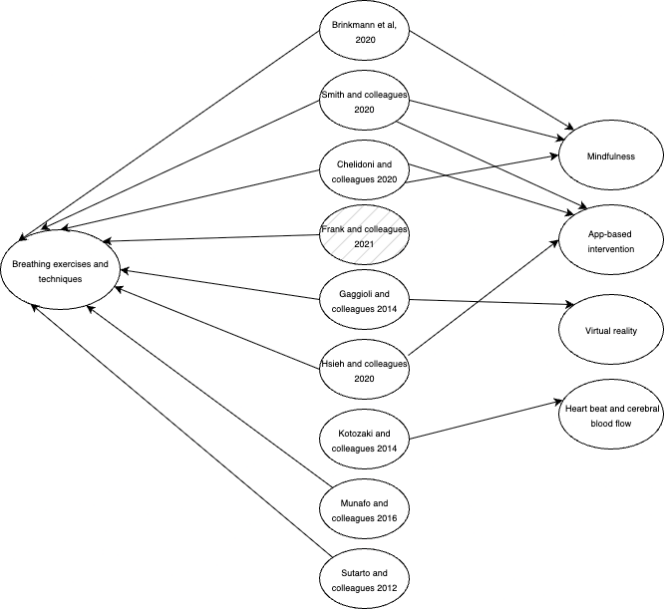
Intervention diagram from the final selected studies.

To visually represent the main interventions identified in our selected studies, we created Figure 3, which demonstrates the relationships among the studies. Of the 9 selected studies, 8 (89%) reported positive results; the remaining study initially showed an increase in stress levels. Breathing exercises and techniques combined with biofeedback were the predominant interventions, providing a simple method to reduce HR variability (HRV) and improve stress levels from both physiological and psychological perspectives.

**Table 1:** Selected studies (N=9).

Study	Sample	Intervention	Questionnaires	Physiological parameters	Results	Follow-up
Sutarto et al [47], 2012	36 workers from a manufacturing company producing electronic parts located in Malaysia (biofeedback: n=19 and control: n=17)	Five sessions of HRV^a^ biofeedback training, 1 session per week. The control group was physically monitored with no feedback instructions with the same cadency.	Trier Inventory for Chronic Stress, Mini-DIPS^b^, stress perception, and coping	HRV parameters: RMSSD^c^, SDNN^d^, and cortisol	High effect size regarding reducing negative emotional symptoms and significant improvements from the baseline follow-up assessment	Time 1: first session Time 2: 1 wk after the final training
Kotozaki et al [48], 2014	30 right-handed workers in Japan (biofeedback: n=15 and control: n=15)	5 min of biofeedback training (joint task of cerebral blood flow and heart rate) every day for 4 wks	CES-D^e^ scale, General Health Questionnaire 30, PANAS^f^ and BJSQ^f^	Cortisol and voxel-based morphometry	Significant decrease in clinical stress outputs after the intervention (CES-D and PANAS-NA^g^), as well as a decrease in work stressors score	Time 1: first session Time 2: 4 wks after the start of the intervention
Gaggioli et al [49], 2014	61 high school teachers recruited in Milan; 60 pediatric nurses from Messina, Italy	(1) Experimental group: n=40; B1=20, B2=20, that received a 5 wk (2 sessions per week) treatment based on the Interreality paradigm; (2) control group: n=42; B1=22, B2=20. 5-wk (2 sessions per week) traditional stress management training based on CBT^h^; and (3) the waitlist group: n=39, B1=19, B2=20, that was reassessed and compared with the 2 other groups 5 wks after the initial evaluation.	VAS-A^i^, STAI-Y1^j^, COPE^k^ Inventory, PSS^l^, PSM^m^, SWLS^n^, and VAS-A	Heart rate and HRV	“Interreality” intervention significantly reduced anxiety, with a large effect size, with this being the primary outcome of the study. The stress outcomes also reduced when compared to the control group.	Time 1: session 1 Time 2: session 10
Munafò et al [50], 2016	31 managers from a private banking firm from the northeast of Italy (biofeedback: n=16 and control: n=15)	Each week, there were five 45-min sessions. Participants were given biofeedback instructions, and the advice was to synchronize heart rate variations and abdominal breathing until the 2 signals covariated.	Sociodemographic variables (age and education, health behavior data, weight, height, physical activity, sleep time, family history of hypertension and cardiovascular disease, and a semistructured interview), STAI-Y—STAI-Y2, and SF-36^o^	Photoplethysmography, respiration rate, SBP^p^, and DPB^q^; skin conductance level	Medium effect size in SBP^#^ and large effect size in RSA^r^. All the participants reported a reduced heart rate at rest, lower levels of anxiety, better health perception, more energy, less fatigue, and better social functioning after the intervention.	Time 1: 1 wk before the training Time 2: 2 wk after the end of the training
Hsieh et al [51], 2020	135 psychiatry ward nurses in Taiwan (biofeedback: n=49, smartphone-based intervention: n=47, and control: n=39)	Every participant went through a 2-h resilience-building course. The biofeedback training protocol included self-guided progressive muscle relaxation, diaphragmatic breathing, and other types of breathing, all of which were performed in 60-min sessions weekly for 6 wks. The breathing techniques included in the protocol were: (1) diaphragmatic breathing, (2) paced breathing, (3) pursed lips breathing, and (4) RSA biofeedback.	Demographic variables, rehabilitation strength chart. Simplified Health Scale, CES-D, OSI-2^s^, and RS^t^	HRV: SDNN, low frequency, high frequency, respiration rate, and RSA	Significant improvements in depressive symptoms, resilience, and respiration rate. The smartphones-delivered intervention showed significant reductions in occupational stress.	Time 1: wk 0 Time 2: wk 6
Brinkmann et al [52], 2020	69 healthy workers from Germany (biofeedback: n=23, MBIs^u^: n=19, and control: n=27)	Participants were told to practice for half an hour every day and to fill out a daily self-report. Question-answer sessions were held twice (after wks 1 and 3). The trainings were guided by experienced trainers in either HRV-Bfb^v^ or MBI.	SVF-120: Stress-Coping Questionnaire, positive and negative coping strategies, BDI-II^w^, Hamburg Modules for the Assessment of Psychosocial Health in Clinical Practice (HEALTH-49), FFA-14^x^, and Self-compassion Scale	ECG^y^, RSA, HRV, and cortisol	No significant differences between biofeedback and mindfulness-based interventions were found. Both interventions had a significant impact on the clinical outcomes, with a small to medium effect size in the primary outcomes (psychological and physiological parameters of stress: stress perception, coping, HRV parameters, and cortisol).	Time 1: baseline (first session) Time 2: after the program (6 wks) Time 3: 6 wks after the program ended (12 wks)
Chelidoni et al [53], 2020	75 full-time working adults from England (BioBase breathing: n=25, mindfulness body scan: n=25, and control: n=25)	Participants were monitored for a baseline period, followed by a stressor period, which included 2 cognitive tasks and 2 emotion-eliciting film clips. They were then randomly assigned to: BioBase app, mindfulness body scan, or control.	Continuous performance task, neuropsychological performance-based task, Go/No-Go; Emotional stress: 2 emotion-eliciting film clips, FFMQ^z^, Samn-Perelli Fatigue Checklist, Stanford Sleepiness Scale, and VAS^aa^	HRV and breathing rate	HRV was higher at recovery for participants who enrolled in the app-directed intervention against the mindfulness and control groups. Significant pretest-posttest difference was observed within the groups, with a small effect size.	Time 1: baseline in sessions and during cognitive and emotional stress induction tasks. Data collection occurred only during sessions, with no separate pre- and postintervention assessments.
Smith et al [54], 2020	169 office workers from 7 US cities (biofeedback: n=67 and control: n=102)	MBSR^ab^ programs and four wearable-based treatment components: (1) wearing of the device itself, (2) tracking and visualization of past physiological states, (3) visual real-time biofeedback, and (4) real-time notifications on significant and sustained changes of the user’s respiratory patterns.	PSS, MASQ^ac^, CDC^ad^ Healthy Days–Days Anxious, Stress quantification (Likert), CDC Healthy Days–Days Sad or in Poor Mental Health, PANAS, and Engagement and Fidelity measures	Respiratory data severely limited.	After the 4-wk intervention period, the treatment group reported experiencing 15.8% fewer negative instances of stress, 13% fewer distressing symptoms, and 28.2% fewer days feeling anxious or stressed compared to control. There was also marginal evidence that the treatment group reported fewer negative emotions, but there was no robust evidence that the intervention increased broad measures of well-being.	Time 1: prestudy survey (1 wk) Time 2: email to download the fully featured app and begin the 4-wk intervention (week 2) Time 3: after the 4-wk intervention, they answered the poststudy survey
Orlando et al [55], 2021	18 health workers from 2 family medicine clinics from California	(Biofeedback: n=9 and control: n=9). 1‑h training session to learn quick‑coherence self‑regulation techniques and practiced using the emWave Pro biofeedback device in the workplace. The biofeedback group was asked to perform 5 mins of daily self‑regulation with optional biofeedback over 12 wks, the first 6 of which included weekly peer support.	Demographic variables, PSS, MSQ‑SF^ae^, and biofeedback minutes	Cardiac Coherence Achievement Score and respiratory data	The treatment group received 1 biofeedback session per week for 6 mins, while the control group had 2 sessions for 11 mins. Perceived stress initially increased in both groups, particularly in the treatment group, but subsequently decreased without statistical significance.	Time 1: baseline Time 2: 6 wks of treatment Time 3: 12 wks of treatment

^a^HRV: heart rate variability.

^b^Mini-DIPS: depression, anxiety, and stress scale.

^c^RMSSD: root mean square of successive differences between normal heartbeats.

^d^SDNN: SD of NN intervals.

^e^CES-D: Center for Epidemiologic Studies Depression.

^f^PANAS: Positive and Negative Affect Schedule.

^g^BJSQ: Brief Job Stress Questionnaire.

^h^CBT: cognitive behavioral therapy.

^i^VAS-A: Visual Analog Scale for Anxiety.

^j^STAI-Y1: State-Trait Anxiety Inventory Form Y-1.

^k^COPE: Coping Orientation to the Problems Experienced.

^l^PSS: Perceived Stress Scale.

^m^PSM: Psychological Stress Measure.

^n^SWLS: Satisfaction with Life Scale.

^o^SF-36: Short Form Health Survey.

^p^SBP: systolic blood pressure.

^q^DBP: diastolic blood pressure.

^r^RSA: respiratory sinus arrhythmia.

^s^OSI-2: Occupational Stress Indicator-2.

^t^RS: Resilience Scale.

^u^MBI: mindfulness-based intervention.

^v^HRV-Bfb: heart rate variability-biofeedback.

^w^BDI-II: Beck-Depression Inventory.

^x^FFA-14: Freiburg Mindfulness Inventory.

^y^ECG: electrocardiogram.

^z^FFMQ: Five Factor Mindfulness Questionnaire.

^aa^VAS: visual analog scale.

^ab^MBSR: mindfulness-based stress reduction.

^ac^MASQ: Mood & Anxiety Symptoms Questionnaire.

^ad^CDC: Centers for Disease Control and Prevention.

^ae^MSQ-SF: Minnesota Satisfaction Questionnaire-Short Form.

### Biofeedback Interventions With Breathing Techniques and Training and Respiratory Sinus Arrhythmia Measures

Among the 9 selected studies, a common approach was the use of breathing interventions and techniques to lower the breathing and HR (Figure 3). These techniques were delivered through different interventions, such as traditional biofeedback and smartphone-delivered biofeedback training.

Munafò et al [50] focused on synchronizing HR and abdominal respiration variations to maximize respiratory sinus arrhythmia (RSA), with participants receiving real-time feedback and guidance through visual displays. All participants in the study reported reduced resting HR, lower anxiety levels, improved health perception, increased energy, decreased fatigue, and enhanced social functioning after the intervention. Importantly, in comparison to the control group (same characteristic as the intervention group), those who underwent the biofeedback training showed a significant natural logarithm of RSA (lnRSA) increase and decrease in both systolic blood pressure and logarithm of skin conductance level (logSCL). In addition, participants who underwent the biofeedback training experienced reduction of emotional interferences in work or other regular daily activities. Hsieh et al [51] explored various breathing techniques, including diaphragmatic breathing, paced breathing, pursed lips breathing, and RSA biofeedback. Brinkmann et al [52] used a mobile HRV training device equipped with an LED light and a blue breathing frequency indicator to guide participants in maximizing their HRV. The device provided real-time feedback and visual cues to synchronize breathing with the desired pattern [52]. This study demonstrated a significant impact on clinical outcomes, showing small to medium effect sizes across various psychological and physiological stress parameters, including stress perception, coping, HRV, and cortisol levels, in both proposed interventions. Smith et al [54] used a wearable device to prime the participants’ awareness and prompt respiratory self-regulation. Chelidoni et al [53] developed a mobile app that delivered guided breathing exercises along with mindfulness body scans. These apps incorporated visual and auditory cues to facilitate specific breathing rates, guiding participants toward optimal HRV levels. Gaggioli et al [49] integrated virtual reality (VR) biofeedback into their intervention, immersing participants in virtual environments while simultaneously guiding them through deep breathing and muscle relaxation techniques. Sutarto et al [47] used a light display that indicated the desired breathing frequency, helping participants synchronize their breath with the intended pattern. This study demonstrates a large effect size in reducing negative emotional symptoms, along with significant improvements from baseline to follow-up assessments. Depression, anxiety, and stress scores were significantly lower in the intervention group compared to the control group. In addition, resonant biofeedback produced a significant decrease in depression (58.3%), anxiety (37.5%), and stress (30.8%) scores in the biofeedback training group from before intervention to after intervention. It is important to note the small and homogeneous sample size, which suggests that these results should be carefully looked upon. Nonetheless, resonant breathing biofeedback training demonstrates promise for the new training approach for reducing negative emotional states.

Among the selected studies, only Orlando et al [55] reported unusual results, indicating a negative impact on physiological outcomes during the initial 6 weeks of the intervention.

### Biofeedback Interventions Using Mobile Apps

Besides focusing on breathing techniques, some mobile apps were reported to be used in 2 (22%) of the 9 selected studies. One of them [53] used a commercial app, BioBase, with guided breathing that has the ability to be modified according to the participants’ HR (physiological data collected through a wearable wrist band). In addition, this app compiles mindfulness body scan and step-by-step instructions, including visual cues to facilitate relaxation and stress reduction. Participants were instructed to perform 5-minute scans, providing nonjudgmental acceptance of thoughts and feelings. Hsieh et al [51] implemented an app-directed self-managed protocol that included a guided meditation practice in the form of an MP4 file. Participants could follow along with the meditation exercises at their own pace, guided by the audio and visual instructions provided through the app [51]. Smith et al [54] presented a commercial app, Spire Health, that allowed participants to visualize respiratory changes over time, observe real-time biofeedback, and receive real-time notifications of physiological stress.

### Biofeedback Interventions Using Mindfulness and Meditation

Overall, the studies analyzed comprised ≥1 intervention technique to provide better comparison between the techniques under study and other well-known stress reduction interventions. Furthermore, 3 studies used mindfulness interventions as a secondary or comparative intervention and 1 study used meditation as a secondary path. Brinkmann et al [52] used traditional mindfulness-based stress reduction techniques with elements of self-compassion, acceptance, commitment, and mindfulness-based cognitive therapy. This formal guided meditation included mindfulness of breathing and mindfulness of thoughts, feelings, and physiological sensations. In addition, informal meditation practices were encouraged using brief pauses throughout the day to focus on present moment awareness without judging. In addition, participants were gifted a meditation CD with 12 guided meditations to support formal meditation at home. The second mindfulness intervention by Chelidoni et al [53] involved using a mobile app that had mindfulness body scans with audio instructions, just like the first one. Participants were encouraged to focus and guide their attention to bodily and breathing sensations, fostering acceptance of thoughts and feelings in the present moment. The study by Hsieh et al [51] defined the intervention as meditation but kept the same principle as the studies before, with the exception of adding visual cues to the auditory stimulus. A video file was used to guide the guide to portrait meditation practice. The researchers tracked and monitored participants’ progress during the training through an in-app cloud service.

### Biofeedback Interventions Using VR and Biosignal Reactive Training

Gaggioli et al [49] used VR experiences to manage stress. Among the selected studies, this is the only one that portraited VR experiences in the workplace. The protocol involved inducing potentially stressful experiences, followed by exposing participants to an immersive natural scenario designed to teach specific relaxation techniques. The training sessions were composed of four stages: (1) homework checking, (2) exposure to a stressful VR environment, (3) relaxation techniques, and (4) homework assignment. The relaxation techniques were induced though the immersion in a natural scenario selected by the participant. These experiences and scenarios are integrated with prerecorded audio narratives with guidance to execute different deep breathing exercises and muscle relaxation techniques. Afterward, the participants are instructed on how to use the smartphone and the body-worn wireless sensor to do the contextualized homework. The interreality intervention significantly reduced anxiety, the primary outcome of the study, with a large effect size.

### Biofeedback Interventions Using Traditional HR Monitoring and Cerebral Blood Flow

Among the selected studies, Kotozaki et al [48] showed a different approach to biofeedback and monitoring, including HR training, cerebral blood flow, saliva sampling, and voxel-based morphometric measures. After the intervention, several clinical stress outputs, including the Center for Epidemiologic Studies Depression Scale, Positive and Negative Affect Schedule–Negative Affect, and Brief Job Stress Questionnaire (BJSQ)-aptitude for job scores, as well as the BJSQ tension, BJSQ depression, and BJSQ stressors of working environment scores, significantly decreased when compared to the control group.

This study introduces a proprietary wearable device that uses near-infrared light at the isosbestic point of oxygenated and deoxygenated hemoglobin to measure concentrations of both oxygen and deoxyhemoglobin in brain tissues, along with HR. The biofeedback sessions were conducted using dedicated software on a PC.

## Discussion

### Principal Findings and Comparison to Prior Work

**Figure 4:**
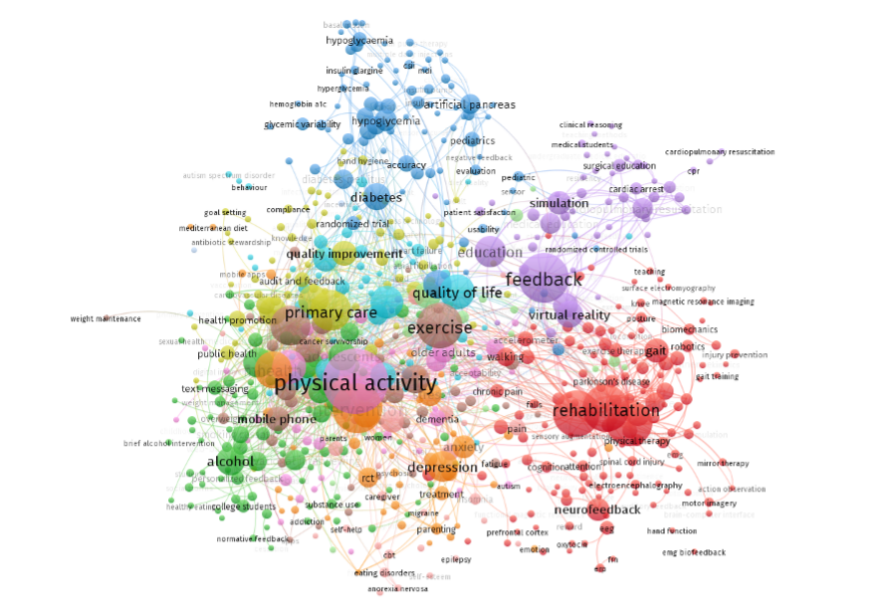
Visualization of the biofeedback publications in the last 13 years.

The results revealed a lack of studies focusing on biofeedback interventions specifically designed to enhance workers’ psychological well-being and mental health. However, numerous studies have explored the use of biofeedback systems in different samples to improve mental health and well-being [56-63]. Despite the limited focus on occupational settings, it is highly relevant to discuss our results in comparison to the most prevalent areas of biofeedback research, namely, physical activity and rehabilitation. To provide a broader context, we created a PubMed citation library of the biofeedback literature using the same keywords as our query. Using VOSviewer 1.6.18, we visualized the obtained results (Figure 4). Notably, despite the intentional inclusion of workplace-related keywords in our research, the term “workplace” does not appear prominently in the resulting diagram, providing an insightful starting point for our visual narrative. The image clearly illustrates an important point: using biofeedback systems to improve mental health at work is an emergent and evolving field that remains relatively unexplored but holds significant potential to contribute to occupational health and safety.

The studies included in our review demonstrated consistent methods of presenting biofeedback information [22,27,64]. Common approaches included the traditional display of HR, HRV, and RSA measures on a screen, as well as the use of visual or auditory cues to indicate the rate of relevant variables. Some studies went further, offering participants an immersive 3D experience that integrated physiological data with the intervention elements. This aligns with findings from other areas of biofeedback research, as most interventions tend to focus on the same methods and variables presented [65-69].

The results showed great potential in different interventions to produce meaningful changes in clinical outcomes. All the selected studies had control groups, with the option to receive intervention later. However, the effects of the intervention were not measured over the medium to long term, and the sample sizes were small. It is also important to address that general biofeedback interventions primarily focus on stress reduction through relaxation training and physiological self-awareness, often without consideration of job-specific stressors. However, workplace interventions should be structured to accommodate work schedules, corporate culture, and industry-specific demands. Given these limitations, the results should be interpreted with caution, with attention focused on the immediate effects of the interventions. For the purpose of this review, we will first focus on studies that demonstrated interesting and promising results.

As indicated in the Results section, breathing techniques were the main intervention in 8 (89%) out of the 9 selected studies. These findings align with previous research on the implementation of breathing techniques for reducing physiological and psychological stress in adults [70-72]. The main differences are the diverse methods used to present or perform biofeedback. We found 5 different methods included in our results: traditional biofeedback, mindfulness, app-directed interventions, immersive scenarios, and in-depth physiological data presentation.

Regarding the comparison between breathing and mindfulness [52], no significant differences were observed between the 2 techniques in terms of their impact on stress-related psychological and physiological parameters, such as stress perception, coping, HRV parameters, and cortisol levels. This information presents a great opportunity to widen the spectrum of available and effective interventions in the workplace to mitigate stress. However, app-directed interventions showed higher HRV at recovery compared to the mindfulness and control groups [53]. It is important to note that this particular study included a stress induction protocol to induce “anger,” identified to induce the highest physiological activation in HR among negative emotions [73]. These results are supported by findings from a study using the same application with a different sample of university students, which reported higher HRV [74]. We also observed notable results for both traditional biofeedback and app-directed interventions, with the latter showing significant improvements in depressive symptoms, resilience, and respiration rate [51]. Concurrently, the app-directed intervention also resulted in significant reductions in occupational stress. It is worth considering that traditional biofeedback can only be provided in a structured environment, such as the workplace, which itself can be a stressful environment. In contrast, app-delivered biofeedback can be carried out during nonworking hours, with very high flexibility and enrolling participants at a time that they may experience less stress, which could explain the observed results. Both groups exhibited a reduction in respiratory rates. Although effect sizes were not reported, significant *P* values were obtained. Similarly, a 2021 study using a smartphone app for HRV measures found that individuals who used the app more frequently reported greater benefits [75]. These results highlight the direction that workplace interventions should take moving forward. Flexibility and ease of use can engage participant adherence and elevate interventions to the next level.

Concerning RSA measures, we observed a large effect size for RSA, skin conductance, and respiratory exchange [50]. In the same line, we encountered very insightful results in resonant breathing biofeedback with a high effect size in reducing negative emotional symptoms and significant improvements from baseline to follow-up assessment. Depression and anxiety were also significantly lower in the intervention group [47]. These results align with previous research on RSA biofeedback and resonant breathing biofeedback, which also reported increases in HRV [76-78].

In addition, it is worth noting that breathing techniques, mindfulness techniques, and app-directed interventions are the easiest, most accessible, convenient, and cost-effective interventions to implement in the workplace. In addition to the existing discussion, it is important to delve deeper into the mechanisms by which breathing techniques facilitate relaxation and stress reduction. Reducing the breathing frequency leads to autonomic changes that increase HRV and RSA, accompanied by modifications in central nervous system activity [79]. An available functional magnetic resonance imaging study highlights heightened activity in the cortical (eg, prefrontal, motor, and parietal cortices) and subcortical (eg, pons, thalamus, subparabrachial nucleus, periaqueductal gray, and hypothalamus) structures [80]. Regarding mindfulness, by leading individuals to focus on moment-by-moment occurrences rather than getting entangled in worry or rumination, mindfulness reduces amygdala activation, leading to decreased overall stress levels [81]. Some studies have identified this sequential pattern of outcomes [82], which may clarify the stronger associations between stress and mindfulness observed in our cross-sectional analyses compared to our longitudinal analyses, where participants’ mindfulness skills were newly acquired at the end of training. Mindfulness practices typically precede a reduction in perceived stress, suggesting that increased trait mindfulness may mediate the link between mindfulness training and stress reduction [83].

With regard to immersive scenarios provided by VR and real-time monitoring, our results showed a significant reduction in anxiety, as the primary outcome, with a large effect size [49]. Despite the interesting results and being able to integrate assessment and treatment within a hybrid environment that bridges the physical and virtual worlds, VR interventions can be costly, requiring expensive hardware, biosensors, and other resources. However, as the industry makes these technologies more affordable, this study stablishes the foundation for further scrutiny in the field. A scoping review on VR biofeedback in 2022 highlighted the importance of these innovative interventions, which tend to have higher adherence rates and offer advantages such as increased motivation, improved user experience, higher engagement, and enhanced attentional focus [84].

Finally, the study by Orlando et al [55] revealed an increase in stress levels during the first 6 weeks of the intervention, while the biofeedback procedure was conducted and supported by investigators. This negative effect was attributed to increased responsibilities and workload associated with daily biofeedback. Notably, this study used self-regulation techniques and limited biofeedback access to desktop-based stations in the workplace, which posed logistical limitations. However, the remaining studies demonstrated positive results in physiological outcomes, showing significant differences between intervention and control groups. Several factors could explain this outcome, including the study design, participant characteristics, or the specific nature of the biofeedback intervention used. However, the additional effort required for participants in the study due to the structured nature of the workplace intervention seems to be a challenge. In this protocol, only 2 desktop-based sets were available, which required participants to allocate different times for the intervention. The small sample size also posed challenges.

This review explored the efficacy and applicability of biofeedback interventions in improving the psychological well-being and mental health of employees within occupational settings and highlights the ones that incorporate visual or auditory cues implanted in breathing techniques. These interventions are convenient, versatile, user-friendly, and potentially cost-effective and capable of reducing stress and improving physiological and psychological well-being among workers and employees with the minimum footprint possible. The diverse range of delivery methods and techniques provide flexibility in the deployment of biofeedback interventions tailored to individual needs and preferences in the workplace.

### Limitations

We want to address some important limitations about this review and the selected studies. The small sample sizes limit the amount of data and generalization we can provide with the outcomes. As this is a rapidly evolving field, a significant increase in studies focused specifically on occupational health and safety may be observed in the coming months. We tried our best to include a very wide range of terms in the search query; however, other terms could come up with different and additional publications. Furthermore, gray literature could also provide interesting reports, although being less desirable. Another limitation of this study is that the database search results varied between the time the original search was performed and a subsequent verification. Although the search strategies were carefully documented, the number of articles retrieved varied, reflecting possible updates or changes in the database indexing. This variability may affect the reproducibility of the initial search results. To address this, we have included a second appendix (Multimedia Appendix 2) showing the search results obtained from a recent search using the same strategy.

The findings of this review highlight the potential of biofeedback interventions in improving workplace well-being across various occupational settings. However, the effectiveness of these interventions may vary by job type and industry-specific demands. For example, interventions involving wearable-based biofeedback and app-based solutions may be more suitable for office workers, who can integrate them into their work routine with minimal disruption. In contrast, structured biofeedback sessions may be more effective in high-stress professions such as health care, where controlled interventions targeting resilience and emotional regulation are essential. Despite these distinctions, none of the included studies explicitly compared intervention effectiveness across different job types. As such, while general improvements in stress reduction, resilience, and physiological regulation were observed, the degree to which intervention success is influenced by occupational factors remains an open question.

### Future Directions

This review may be of interest to researchers, developers of mental health interventions, and practitioners interested in the use of biofeedback in mental health management as it presents descriptions and analysis of 9 examples of biofeedback-based interventions to improve workers’ psychological well-being and mental health, including 5 different breathing techniques, app-based programs intervention, VR, and cerebral blood flow. In addition, the selected studies provide a solid foundation into assessing the interventions feasibility, indicating not only standard questionnaires but also objective physiological measures and indicators.

An important note regarding future challenges for this emergent risk in psychosocial factors is that the adherence is very low. This growth may face challenges from various fronts, with privacy concerns, resource availability, and workplace logistics being key barriers to implementing biofeedback interventions. Employees may be apprehensive about data privacy, employer access to biometric information, and potential misuse, necessitating clear governance policies, anonymization, and secure data storage. In addition, engagement and adherence challenges arise due to time constraints and varying motivation levels, particularly in high-demand work environments. To ensure successful implementation, organizations should prioritize transparency, integrate interventions into existing workflows, and provide adequate education and support to enhance employee acceptance and long-term effectiveness. We crave an effective jump from the laboratorial context to the real work environment. Recent approaches with higher engagement and user experience seem to bump adherence, for example, VR and augmented reality, despite not being the ideal intervention for the work environment. With this in mind, there is a tremendous need for solutions that offer higher engagement and adherence, enable unobtrusive use, and operate independently—functioning as supportive tools rather than adding to the workload. Given the variability in workplace environments, future research should explore how biofeedback interventions can be tailored to specific job demands and occupational settings. Different work environments present unique stressors and logistical challenges that may impact intervention feasibility and effectiveness. Future studies should also evaluate how workplace-specific constraints influence engagement, adherence, and outcomes, ensuring that interventions are not only effective but also practical and accessible for diverse job roles. In addition, research should examine employer and employee perceptions regarding biofeedback adoption in different industries, addressing potential concerns such as usability, data privacy, and workplace acceptance.

These digital systems are the first line of health monitoring in industries and enterprises. Biofeedback systems provide a window into understanding employees’ health and psychophysiological states to improve occupational safety and security and prevent further costs, highly contributing to companies’ sustainability.

In addition, it is very important to address cost-effectiveness of these interventions in the future. However, the cost-effectiveness of biofeedback interventions will vary widely depending on the type of intervention, technological requirements, and workplace setting. Among the different approaches, mindfulness-based and app-directed biofeedback interventions appear to emerge as the most cost-effective solutions. These interventions require minimal investment, are easily scalable, and have demonstrated positive outcomes in stress reduction and HRV recovery. Their affordability and accessibility make them particularly appealing for organizations seeking low-cost mental health interventions with broad employee engagement.

On the other end of the spectrum, VR-based biofeedback and real-time physiological data monitoring represent higher-cost but high-impact interventions. VR biofeedback provides an immersive experience that enhances stress reduction, yet its implementation requires significant investment in hardware, software, and user training. Similarly, real-time physiological monitoring offers highly personalized stress management solutions; however, it requires continued investment in wearable sensors and data processing infrastructure. These approaches may be more suitable for large organizations with dedicated resources for long-term employee well-being initiatives.

Traditional biofeedback, delivered through structured, on-site programs, remains an effective but less flexible option. These interventions often require trained personnel, dedicated equipment, and designated spaces, limiting their accessibility in certain workplace environments. In contrast, self-guided or mobile-based interventions provide greater feasibility and scalability, allowing employees to engage with biofeedback techniques at their convenience.

While none of the selected studies explicitly reported costs or return on investment metrics for biofeedback interventions in workplace settings, understanding the economic impact of such interventions is critical for decision makers. Workplace health promotion programs have been extensively studied in terms of cost-effectiveness, and findings suggest that investing in employee well-being yields significant financial benefits. Evidence from general workplace health programs indicates that for every US $1 invested in workplace health promotion, there is a US $3.27 reduction in medical expenses and a US $2.73 decrease in absenteeism costs [85]. In addition, such programs contribute to annual employer savings of US $15.6 for every US $1 spent, primarily due to increased productivity and reduced absenteeism [86].

Further research on psychosocial safety climate (PSC), which aligns closely with stress management and mental health interventions, highlights additional cost benefits. Enhancing PSC can result in a 43% reduction in sickness absence and a 72% decrease in presenteeism, demonstrating the financial impact of workplace stress reduction strategies. A medium-sized company with 100 employees could save over US $180,000 annually, equating to US $1887 per employee, when transitioning from a low to a high PSC environment [87].

### Conclusions

This review promotes future work in developing and implementing interventions to improve well-being and mental health in workplaces, paving the way for a guided start. Furthermore, biofeedback systems can be enhanced by serving not only as intervention tools but also as early detection systems. The important collaboration with health care professionals and the industries will further strengthen the possibility for positive outbreaks in the workplace, and soon, we will have better, safer, more sustainable, and healthier workplaces. By implementing these interventions and fostering a culture that values mental well-being, we can transform the workplace into a space where employees thrive, creativity flourishes, and productivity soars. It is time to acknowledge the importance of mental health and take bold steps toward creating a healthier and happier workforce. After all, our collective success depends on the well-being of each individual.

By addressing mental health issues proactively, these interventions contribute significantly to the broader objectives of safety science. First, enhancing mental well-being through biofeedback directly correlates with reduced workplace accidents, as a mentally healthy workforce is more alert, aware, and able to respond to potential hazards effectively. Second, the improvement in employee mental health is inherently linked to increased productivity. Workers with better mental health are more engaged, motivated, and capable of performing tasks efficiently, driving organizational performance and growth. Ultimately, fostering a safer work environment through biofeedback interventions aligns with the strategic goals of occupational health initiatives. As mental health challenges in the workplace receive increasing recognition, integrating biofeedback into routine employee wellness programs becomes instrumental. These interventions act as preventive measures, reducing the incidence of work-related stress, burnout, and psychosocial risks. Importantly, they not only ensure the individual well-being of employees but also contribute to a culture of safety, resilience, and sustainability within organizations.

## Appendix

Multimedia Appendix 1 Database and search queries used in this study.

Multimedia Appendix 2 Database and search queries used in this study (recent search).

Multimedia Appendix 3 PRISMA checklist.

## Abbreviations

BJSQ: Brief Job Stress Questionnaire
CONSORT-EHEALTH: Consolidated Standards of Reporting Trials of Electronic and Mobile Health Applications and Online Telehealth
EU: European Union
HR: heart rate
HRV: heart rate variability
PEDro: Physiotherapy Evidence Database
PRISMA: Preferred Reporting Items for Systematic Reviews and Meta-Analyses
PSC: psychosocial safety climate
RSA: respiratory sinus arrhythmia
VR: virtual reality
