# Supplementary material for: Interventions Based on Biofeedback Systems to Improve Workers’ Psychological Well-Being, Mental Health, and Safety: Systematic Literature Review
Source: J Med Internet Res. 2025 Sep 12;27:e70134. doi: 10.2196/70134 (PMC12475886; doi:10.2196/70134)
Supplement: Multimedia Appendix 1 [file jmir_v27i1e70134_app1.docx]

**Multimedia Appendix 1**

Database and search queries used in this study.

| **DB** | **Topic** | **Query** | **Results** |
| --- | --- | --- | --- |
| PubMed | Mental health and job performance  AND  [1] | “fatigue”[MeSH] OR “lassitude”[All fields] OR “mental health”[MeSH] OR “mental disorders”[MeSH] OR “stress”[All fields] OR “mood disorders”[MeSH] OR “behavioral symptoms”[MeSH] OR “anxiety”[MeSH] OR “burnout”[All fields] OR “absenteeism”[MeSH] OR “Job performance”[All fields] OR “Performance at Work”[All fields] OR “productivity”[All fields] OR “efficiency”[MeSH] OR “Occupational Stress”[MeSH] OR “Job satisfaction”[MeSH] OR “Quality of working life”[All fields] | 889,011 |
|  | Biofeedback  AND  [2] | “biofeedback”[All fields] OR “bio-feedback”[All fields] OR “feedback”[All fields] OR “wearable”[All fields] OR “wearable electronic devices”[MeSH] OR “monitoring, physiological”[All fields] OR “clinical alarms”[MeSH] OR “outcome measures”[All fields] OR “real time”[All fields] OR “self-monitoring”[All fields] | 326,428 |
|  | Workplace  [3] | “job”[All fields] OR “job site”[All fields] OR “workplace”[MeSH] OR “work place” [All fields] OR “worker”[All fields] OR “employee”[All fields] OR “occupation”[All fields] OR “operators”[All fields] or “Occupational”[All fields] | 205,071 |
|  | Together [4] | #1 AND #2 AND #2 | 2009 |
| B.ON  EBSCO | Mental health and job performance  AND  [1] | SU (“fatigue” OR “lassitude” OR “mental health” OR “mental disorders” OR “stress” OR “mood disorders” OR “behavioral symptoms” OR “anxiety” OR “burnout” OR “absenteeism” OR “Job performance” OR “Performance at Work” OR “productivity” OR “efficiency” OR “Occupational Stress” OR “Job satisfaction” OR “Quality of working life”) | 2,332,079 |
|  | Biofeedback  AND  [2] | SU (“biofeedback” OR “bio-feedback” OR “feedback” OR “wearable” OR “wearable electronic devices” OR “monitoring, physiological” OR “clinical alarms” OR “outcome measures” OR “real time” OR “self-monitoring”) | 410,756 |
|  | Workplace  [3] | SU (“job” OR “job site” OR “workplace” OR “work place” OR “worker” OR “employee” OR “occupation” OR “operators” or “Occupational”) | 622,329 |
|  | Together [4] | #1 AND #2 AND #2 | 861 |
