# Supplementary material for: Interventions Based on Biofeedback Systems to Improve Workers’ Psychological Well-Being, Mental Health, and Safety: Systematic Literature Review
Source: J Med Internet Res. 2025 Sep 12;27:e70134. doi: 10.2196/70134 (PMC12475886; doi:10.2196/70134)
Supplement: Multimedia Appendix 2 [file jmir_v27i1e70134_app2.docx]

**Multimedia Appendix 2**

Database and search queries used in this study (recent search).

| **DB** | **Topic** | **Query** | **Results** |
| --- | --- | --- | --- |
| PubMed | Mental health and job performance  AND  [1] | “fatigue”[MeSH] OR “lassitude”[All fields] OR “mental health”[MeSH] OR “mental disorders”[MeSH] OR “stress”[All fields] OR “mood disorders”[MeSH] OR “behavioral symptoms”[MeSH] OR “anxiety”[MeSH] OR “burnout”[All fields] OR “absenteeism”[MeSH] OR “Job performance”[All fields] OR “Performance at Work”[All fields] OR “productivity”[All fields] OR “efficiency”[MeSH] OR “Occupational Stress”[MeSH] OR “Job satisfaction”[MeSH] OR “Quality of working life”[All fields]  Filters applied: Abstract, Full text, English, Humans, Exclude preprints, MEDLINE, from 2012/3/11 - 2024/3/11. | 889,681 |
|  | Biofeedback  AND  [2] | “biofeedback”[All fields] OR “bio-feedback”[All fields] OR “feedback”[All fields] OR “wearable”[All fields] OR “wearable electronic devices”[MeSH] OR “monitoring, physiological”[All fields] OR “clinical alarms”[MeSH] OR “outcome measures”[All fields] OR “real time”[All fields] OR “self-monitoring”[All fields]  Filters applied: Abstract, Full text, English, Humans, Exclude preprints, MEDLINE, from 2012/3/11 - 2024/3/11. | 326,472 |
|  | Workplace  [3] | “job”[All fields] OR “job site”[All fields] OR “workplace”[MeSH] OR “work place” [All fields] OR “worker”[All fields] OR “employee”[All fields] OR “occupation”[All fields] OR “operators”[All fields] or “Occupational”[All fields]  Filters applied: Abstract, Full text, English, Humans, Exclude preprints, MEDLINE, from 2012/3/11 - 2024/3/11. | 205,115 |
|  | Together [4] | #1 AND #2 AND #3  Filters applied: Abstract, Full text, English, Humans, Exclude preprints, MEDLINE, from 2012/3/11 - 2024/3/11. | 2633 |
| B.ON  EBSCO | Mental health and job performance  AND  [1] | SU (“fatigue” OR “lassitude” OR “mental health” OR “mental disorders” OR “stress” OR “mood disorders” OR “behavioral symptoms” OR “anxiety” OR “burnout” OR “absenteeism” OR “Job performance” OR “Performance at Work” OR “productivity” OR “efficiency” OR “Occupational Stress” OR “Job satisfaction” OR “Quality of working life”)  Filters applied: Full Text via Editor, Scientific Journals (Peer Reviewed), Available in Library Collection | 2 338 318 |
|  | Biofeedback  AND  [2] | SU (“biofeedback” OR “bio-feedback” OR “feedback” OR “wearable” OR “wearable electronic devices” OR “monitoring, physiological” OR “clinical alarms” OR “outcome measures” OR “real time” OR “self-monitoring”)  Filters applied: Full Text via Editor, Scientific Journals (Peer Reviewed), Available in Library Collection | 411 612 |
|  | Workplace  [3] | SU (“job” OR “job site” OR “workplace” OR “work place” OR “worker” OR “employee” OR “occupation” OR “operators” or “Occupational”)  Filters applied: Full Text via Editor, Scientific Journals (Peer Reviewed), Available in Library Collection | 624 411 |
|  | Together [4] | #1 AND #2 AND #3  Filters applied: Full Text via Editor, Scientific Journals (Peer Reviewed), Available in Library Collection | 773 |
